# Evaluation of the organic and functional results of tympanoplasties through a retro-auricular approach at a medical residency unit

**DOI:** 10.1590/S1808-86942011000200013

**Published:** 2015-10-19

**Authors:** José Carlos Bolini de Lima, Silvio Antonio Monteiro Marone, Oswaldo Martucci, Fabiana Gonçalez, João Jovino da Silva Neto, Alice Carolina Mataruco Ramos

**Affiliations:** 1 Otorhinolaryngology specialist, ABORL-CCF. Otorhinolaryngologist; 2 Doctoral degree in otorhinolaryngology, Sao Paulo University Medical School. Full professor of otorhinolaryngology at the Medical School of the Pontifical Catholic University in Campinas, SP. Preceptor at the Otorhinus Clinic and the Santa Marcelina Hospital; 3 Otorhinolaryngology specialist, ABORL-CCF. Preceptor at the Otorhinus Clinic and the Santa Marcelina Hospital; 4 Master's degree in otorhinolaryngology, Holy House of Mercy (Santa Casa de Misericórdia), Sao Paulo. Preceptor at the Otorhinus Clinic; 5 Otorhinolaryngology specialist, ABORL-CCF. Otorhinolaryngologist; 6 Otorhinolaryngology specialist, ABORL-CCF. Otorhinolaryngologist. Otorhinus Clinic (Clínica Otorhinus)

**Keywords:** otitis media, hearing loss, tympanoplasty, tinnitus

## Abstract

Tympanoplasty aims at rebuilding the tympanic membrane with or without middle ear functional recovery.

**Aim:**

To evaluate the surgical results of tympanoplasties with a retro-auricular surgical approach at a medical residency unit.

**Materials and Methods:**

Thirty-nine patients with diagnosis of simple chronic otitis media were evaluated; these patients underwent tymplanoplasty by a retro-auricular approach (underlay technique) at a medical residency unit. Patients were included in a prospective medical and audiologic investigation protocol that consisted of a clinical, otomicroscopic and audiometric evaluation. All procedures were supervised by training specialists otorrinolaringology.

**Results:**

The rate of surgical success - full integration of the graft - was 95% of cases. Improvement of hearing, as demonstrated in audiometry, occurred in 72% of cases. Improvement in tinnitus was demonstrated subjectively on a visual analog scale in 69% of cases.

**Conclusion:**

Tympanoplasty through a retro-auricular approach is easy to perform. Full graft integration occurred in 95% of cases and was independent of factors deemed by many authors as relevant. The results - improvement of the quality of hearing and tinnitus - were significant.

## INTRODUCTION

Tympanic membrane perforations are seen often in daily clinical practice, and results from events such as: otologic infection, trauma, or after placing grommets.

The main symptoms in patients with chronic otitis media (COM) alone are hypoacusis and intermittent otorrhea, which are generally associated with upper airway infection or extrinsic contamination by water.

Tympanoplasties are surgical procedures that aim to treat the complications of COM. The aim is to reconstruct the perforated tympanic membrane and to inspect or recover middle ear function. These procedures are indicated if tympanic perforation has persisted for more than three months with or without hearing impairment.^1^

The surgical approach for tympanoplasties may be endoaural or transmeatic, retroauricular (Wilde), and suprameatal (Lempert).^1^ The most common grafting techniques are the underlay (medial) and the overlay (lateral). Temporal muscle fascia and tragus perichondrium cartilage are the most frequently used grafts.^2^, ^3^

The literature mentions several factors that may affect surgical results, including age, perforation size, the site of perforation, the status of the opposite ear, smoking, the state of the middle ear mucosa, tympanosclerotic plaques, and the type of graft.

A few authors have added surgical experience as a factor affecting results.^4^, ^5^, ^6^ Vartiainen (1998)^6^ showed that the tympanic membrane healing rate was 78% when operated by residents, and 95% when operated by experienced surgeons. Fukuchi et al. (2006)^5^ concluded that low grafting success rates (65%) in their study was because the procedures were carried out by second year medical residents.

Other papers, however, have stated that surgical experience did not affect the results.^7^

## OBJECTIVE

The purpose of this study was to assess prospectively the results of retroauricular tympanoplasties undertaken by medical residents, focusing on: 1) graft integration; 2) subjective auditory behavior relative to tinnitus and hearing quality, and objective auditory behavior relative to audiometric results; and 3) an analysis of factors that may have influenced the success of surgery.

## MATERIALS AND METHODS

The research institutional review board of the Otorhinus Clinic, SP approved this study (protocol no. 81/09.

The series comprised patients seen at the Otorhinus Clinic, SP, diagnosed with chronic otitis media alone, which underwent tympanoplasty carried out by otorhinolaryngology medical residents from January to October 2009.

### Definition of the sample

The inclusion criteria were:

1 signing a free informed consent form (ABORL) after being informed about the upcoming procedures;
2 age over 10 years;
3 a diagnosis suggesting COM alone;
4 indication of tympanoplasty in the affected ear;
5 patient wishing to undergo surgery.

The exclusion criteria were:

1 disagreeing with the free informed consent form (ABORL) after being informed about the upcoming procedures.
2 age below 10 years;
3 chronic suppurative otitis media, COM and cholesteatoma, or COM alone that reacutized within the past three months;
4 systemic diseases contraindicating surgery;
5 refusing surgery.

Tympanoplasties were carried out from January to October 2009 by third year otorhinolaryngology residents at the Otorhinus Clinic, SP, which has an ABORL-CCF-accredited residency program under the supervision of a medical preceptor, an otorhinolaryngological surgeon.

All procedures were done under general anesthesia. A retroauricular approach was used. The underlay or medial technique was used for placing the graft. Temporal muscle fascia and tragus perichondrium cartilage were the chosen grafts. If the ossicular chain required reconstruction, temporal bone cortex fragments or ossicular chain interposition was used.

The medical and audiological evaluation protocols were applied seven days before and 60 days after surgery, by medical doctors and speech therapists at the Clinic.

The postoperative assessment of graft integration was done on the 2^nd^, 6^th^, 10^th^, 14^th^, 20^th^, 30^th^, 45^th^, and 60^th^ days by means of an otomicroscopic examination by the medical resident that operated the case, supervised by an assistant physician (preceptor).

Pure tone audiometry and voice audiometry at 250, 500, 1000, 2000, 3000, 4000, 6000, and 8000 Hz was done preoperatively if there was no otorrhea for at least three months; the same tests were done 60 days postoperatively in all patients.

**Figure 1:**
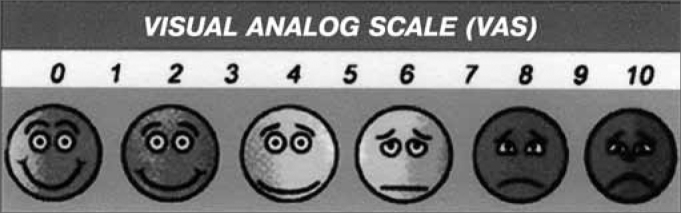
Model of the visual analog scale (VAS) used in this study.

A visual analog scale (VAS) was used as a subjective method for evaluating and measuring tinnitus, as most patients understand this approach. Patients scored their tinnitus from 0 to 10, using a ruler where the lowest scores correspond to the highest satisfaction levels. Thus, score 0 was absent tinnitus, scores 1 to 3 were mild tinnitus, scores 4 to 7 were moderate tinnitus, and scores 8 to 10 were severe tinnitus (Fig. 1).

### Statistical analysis

The statistical analysis was done using Student's t test for the comparison of differences among groups on graft integration (Table 1). Logistic regression was applied to examine the relationship between graft success or failure, and possible independent variables (Table 2). Significant values were *p*<0.05.

**Table 1:** Mean value of factors that may affect graft integration.

	Graft integrated	Graft not integrated	*p* statistics
Preoperative gap	27.2 3	28.25	0.760
Postoperative gap	7.34	27.19	*0.001
Preoperative tinnitus	4.60	4.70	0.640
Postoperative tinnitus	1.40	4.50	*0.002
Size of tympanic perforation (%)	49.29	56.34	0.432
Age	31.43	30.51	0.216

* statistically significant values in the t test (p<0.05).

**Table 2:** Graft integration or not relatie to several parameters (total percentage).

	Graft integrated	Graft not integrated	*p* statistics
Smoker	100% (8 pcts)	0%	0.626
Non-smoker	93.5% (29pcts de 31pcts)	6.5% (2 pcts de 31 pcts)	0.435
Left ear perforation	100% (16 pcts)	0%	0.542
Right ear perforation	91.3% (21 pcts de 23 pcts)	8.7% (2 pcts de 23 pcts)	0.468
Perforation in anterior quadrants	96% (24 pcts de 25 pcts)	4% (1 pct de 25 pcts)	0.430
Perforation in posterior quadrants	92.8%(13 pcts de 14 pcts)	7.2%(1 pct de 14 pcts)	0.348
Normal contralateral ear	93.9% (31 pcts de33 pcts)	6.1% (2pcts de 33 pcts)	0.452
Diseased contralateral ear	100% (6 pcts)	0%	0.640
Middle ear mucosa - dry	94.5% (35 pcts de 37 pcts)	5.5% (2 pcts de 37 pcts	0.254
Middle ear mucosa - wet/hyperplastic	100% (2 pcts de 2 pcts)	0%	0.387
Tympanosclerotic plaques	92.3% (12 pcts de 13 pcts)	7.7% (1 pct de 13 pcts)	0.412
No tympanosclerotic plaques	96.1% (25 pcts de 26 pcts)	3.9% (1 pct de 26 pcts)	0.456
Temporal fascia	94.2% (33 pcts de 35 pcts)	5.8% (2 pcts de 35 pcts)	0.231
Tragus perichondrium	100% (4pcts)	0%	0.431

^*^Independent variables with statistical significance in relation to graft integration (p<0.05).

## RESULTS

Several factors were noted that might have affected the outcome of surgery in 39 patients undergoing tympanoplasty, as shown below (Tables 1 and 2).

The minimal age of patients undergoing tympanoplasty was 10 years and the maximal age was 69 years. The mean age was 30.3 years (standard deviation ± 13.5 years). There were 20 female patients (51%) and 19 male patients (49%). Eight patients (21%) had a history of smoking for over 5 years; the remaining patients (31 cases, 79%) did not smoke.

The perforation was on the right in 23 patients (59%), and on the left in 16 patients (41%).

The perforation sites were the antero-inferior quadrant (24 patients, 61%), the postero-inferior quadrant (12 patients, 31%), the postero-superior quadrant (2 patients, 5%), and the antero-superior quadrant (1 patient, 3%).

The perforation size in operated ears ranged from 10% to 90%. The mean perforation size was 52.9 ± 12 %. There were perforations with areas over 50% in 24 cases (62%).

Six patients (15%) presented sequelae of COM alone in the contralateral ear. Most patients (33 cases, 85%) had normal contralateral ears.

We used temporal muscle fascia in 35 patients (90%), and tragus perichondrium cartilage in 4 patients (10%).

A wet mucosa (mild hyperplasia) was observed perioperatively in 2 patients (5.1%). Tympanosclerotic plaques were seen in 13 patients (33.3%); a disarticulated ossicular chain was noted in 4 patients (10.2%).

Postoperative complications were observed in 8 patients (20.5%). Three of these (7.6%) developed local infection, 2 patients (5.1%) had local infection and late facial paresthesia (fifth postoperative day), 1 patient (2.5%) had facial paresthesia, 1 patient (2.5%) had facial paresthesia and dysgeusia, and 1 patient (2.5%) had dysgeusia.

Grafts were integrated successfully in 37 patients (95%). Temporal muscle fascia was used in 35 patients; of these, the graft was successful in 33 cases (94.2%). Tragus perichondrium was used in 4 patients, and all grafts were successful (100%).

**Chart 1:**
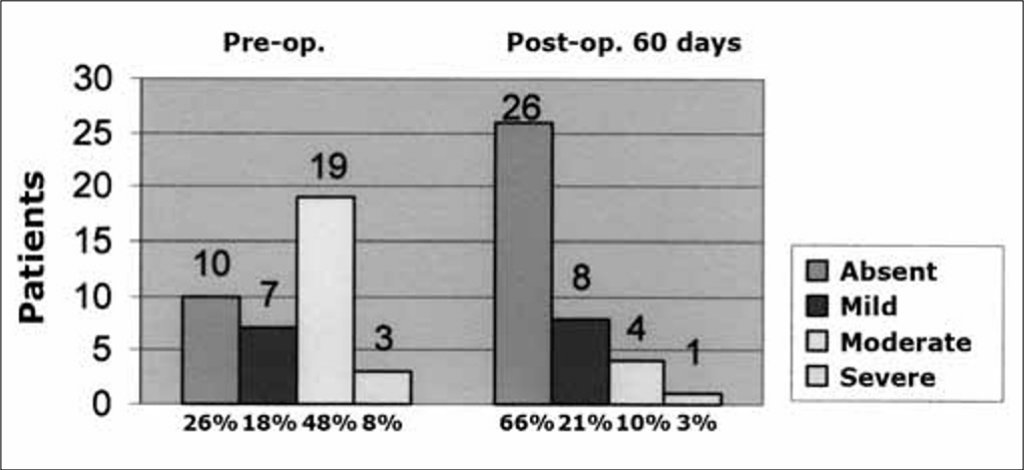
Answer to the question asked before and after surgery (60 days postoperatively): How to you classify your discomfort with tinnitus? Scores on the numeric VAS (score 0 - absent tinnitus, scores 1 to 3 - mild tinnitus, scores 4 to 7 - moderate tinnitus, and scores 8 to 10 - severe tinnitus).

The VAS was applied preoperatively and 60 days postoperatively for a subjective evaluation of tinnitus. Preoperatively, 19 patients (48%) had moderate tinnitus (mean score - 6.7 points), 10 patients (26%) had no tinnitus (score 0), 7 patients (18%) had mild tinnitus (mean score - 3.1 points), and 3 patients (8%) had severe tinnitus (mean score - 9 points). Postoperatively, we found that 26 patients (66%) had no tinnitus (score 0), 8 patients (21%) had mild tinnitus (mean score - 2.9 points), 4 patients (10%) had moderate tinnitus (mean score - 6.1 points), and only 1 patient (3%) had severe tinnitus (mean score - 10) (Chart 1).

Tinnitus improved in 17 patients (43%), regressed in 10 patients (26%), and it remained unaltered in 12 patients (31%); tinnitus did not worsen in any of the cases.

**Chart 2:**
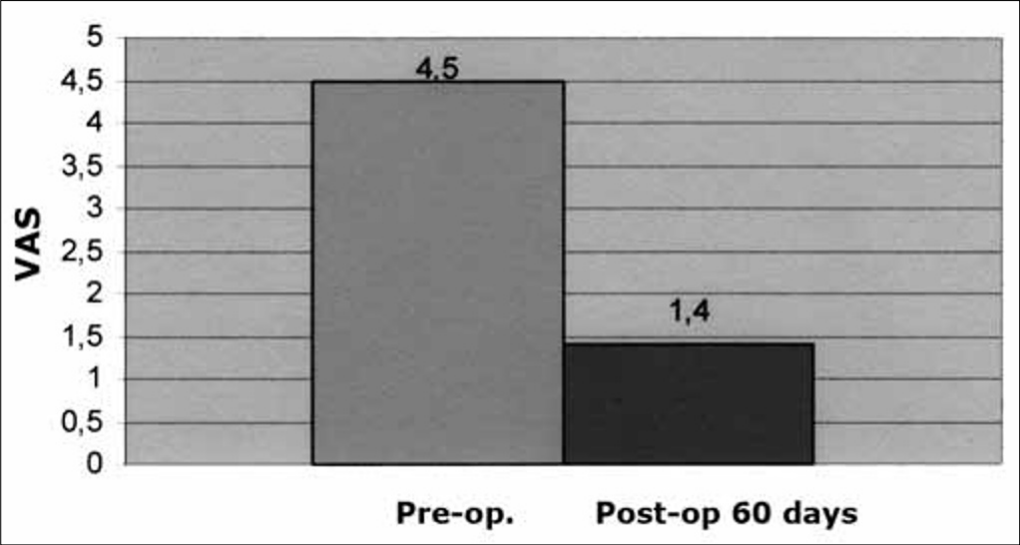
Mean values in the numeric VAS (scores 0 to 10) before and after surgery (60 days postoperatively) in answer to the question: How do you classify you discomfort with tinnitus?

The mean preoperative score on the VAS was 4.5 (moderate tinnitus); the mean postoperative score on the VAS was 1.4 (mild tinnitus) (Chart 2).

Hearing improved in most patients (28 cases, 72%). It did not change in 11 patients (28%), and did not worsen in any of the cases.

**Chart 3:**
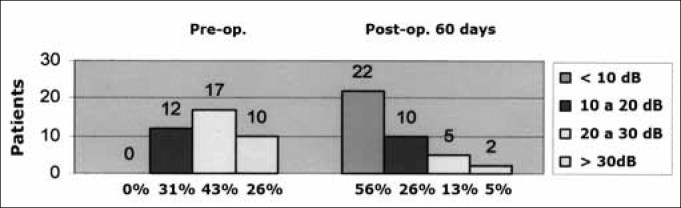
Audiometry of the air-bone gap before and after surgery (60th postoperative day).

Preoperatively, we found that all patients (39 cases, 100%) had conductive hearing loss (in line with the clinical picture of COM alone). Most patients (18 cases, 46%) had moderate hearing loss before surgery. The remaining patients had mild conductive hearing loss (11 cases, 28%) or severe conductive hearing loss (10 cases, 26%). The minimal initial air-bone gap was 5 dB, going up to 50 dB (mean - 27.1 dB). Seventeen of these cases (43%) had air-bone gaps from 20 to 30 dB; 12 cases (31%) had air-bone gaps from 10 to 20 dB, and 10 cases (26%) had an air-bone gap over 30 dB (Chart 3).

On the last postoperative assessment (60^th^ day) we found that the minimum air-bone gap was 0 dB and the maximum air-bone gap was 35 dB (mean - 10.3 dB). The gap closed in 22 patients (56%). It remained similar or decreased in the remaining cases: 10 patients (26%) had a gap from 10 to 20 dB, 5 patients (13%) had a gap from 20 to 30 dB, and only 2 patients had a gap over 30dB (mean - 7.3 dB (Chart 3).

A study of the speech recognition threshold (SRT) showed that the mean postoperative difference was 18.3 dB. The preoperative mean was 41.4 dB, going to 23.1 dB postoperatively.

## DISCUSSION

Measures of results that most often define the success of tympanoplasty are graft integration, an aerated ear, disease control, and the results of hearing.^6^The literature diverges about the surgical success rate and the best technique.

Angeli et al. (2006)^8^ used an overlay technique in a study showing a 91% success rate. Hung at al. (2004)^9^ used an underlay technique to reach an 85.7% graft integration rate in a pediatric population, and 82.4% in adults. Fishman et al. (2005)^10^ reached an overall success rate of 97% using temporal fascia to reconstruct the tympanic membrane.

Because there was no consensus about which technique was best, Rizer (1997)^11^ compared the overlay and underlay techniques. This study comprised 709 tympanoplasties, of which 551 were done with the underlay technique and 158 with the overlay technique; all used temporal fascia as the graft. The success rate - graft integration - was 88% (overlay) and 95.6% (underlay). Similarly, Bastos Freitas (2000)^12^ had graft integration rates of 90.6% (overlay) and 87.9% (underlay).

Jung & Park (2005)^13^ combined both techniques (medial-lateral or underlay-overlay technique) to reach a 97% success rate.

Our success rate (integrated graft) was 95%. This result is considered high when compared to other published means, especially when taking into account that the procedures were done by medical residents. Fukuchi et al. (2006)^5^ and Vartiainen (1998)^6^ concluded that low success rates for graft integration (65% and 78% respectively) were because the procedures were done by medical residents.

Our high success rates are probably due to a systematic surgical technique and the presence of an experienced preceptor during all of the procedures. We also attributed our success rate to the ease of this technique and the fact that complications are infrequent. This technique also offers an excellent view, which allows easy access to the borders of the tympanic perforation and middle ear structures, increasing the safety and facilitating removal of middle ear diseases, removing adhesions, accommodating the graft adequately, and rebuilding the ossicular chain, all of which are essential for surgical success.

The aim of tympanoplasty is also to provide audiometric gains. McGrew et al. (2004)^14^ showed that 76 patients (36%) closed the gap by up to 10 dB HL, and 74 patients (34%) attained a final gap around 11 to 20 dB HL. Lima et al. (2007)^15^ attained values of 61% and 22%. We attained gap closures of up to 10 dB in 22 patients (56%) and a final gap from 11 to 20 dB HL in 10 patients (26%), results that were better than those of McGrew et al. and similar to those of Lima et al. This difference with McGrew et al.'s study may have been because it was a retrospective study. The similarity with Lima et al.'s results may be explained by the fact that is was also a prospective study with a similar method.

In our study, the mean preoperative air-bone gap decreased from 27.1 dB HL to 10.3 dB HL postoperatively, resulting in significant gains in the SRT. This went from 41.4 dB preoperatively to 23.1 dB postoperatively.

Based on these results, we concluded that there was a statistically significant improvement in the quality of hearing of patients undergoing tympanoplasty (*p*=0.002). We believe this improvement was due to graft integration and better conditions in the middle ear and ossicular chain as a result of surgery.

The VAS was applied in measuring tinnitus subjectively; we found that 26 patients (66%) had mild to moderate tinnitus preoperatively, compared with only 12 patients (31%) postoperatively. In our study, tinnitus improved in 17 patients (43%), it disappeared in 10 patients (26%), and remained unaltered in 12 patients; it did not worsen in any of the cases. The preoperative mean in the VAS was 4.5 (moderate tinnitus and the postoperative mean was 1.4 (mild tinnitus).

Thus, we concluded that there was a statistically significant improvement in tinnitus in patients undergoing tympanoplasty (*p*=0.002). We believe that this improvement was the result of graft integration and better conditions in the middle ear and ossicular chain because of surgery.

The literature mentions several factors that may affect surgical results; these include: age, perforation size, perforation site, the status of the opposite ear, smoking, the state of the middle ear mucosa, tympanosclerotic plaques, and graft type. However, there is no consensus, and papers diverge in their results.

A few authors have reported that graft integration is less successful in children than in adults.^16^, ^17^ Other consider that age does not affect surgical results.^6^, ^8^ Age was not statistically significant for success in our study (*p*=0.216).

The perforation size is also an important prognostic variable according to some authors. The main limitations are difficulty in attained adequate exposure of the borders of the perforation, and poorer blood perfusion. These authors have reported that graft integration rates if perforations are large may be only 56%.^18^, ^19^ In our study, we had perforations with over 50% of the tympanic area (mean - 52.9 ± 12%). The mean perforation size in patients where grafts became integrated was 49.29%; it was 56.29% in the patients where the graft did not integrate. These results demonstrated that the perforation size was not statistically significant for surgical success (*p*=0.432).

Other authors have mentioned that the perforation site influences the prognosis more than the perforation size.^20^, ^21^ Anterior perforations are technically more difficult to access and to place a graft adequately; the blood supply is also poorer. Singh et al. (2005)^22^ showed that the graft integration rate was 34% in anterior perforations, 91% in inferior perforations, and 100% in posterior perforations. Other studies have concluded that the perforation site had no effect on graft integration or the results of hearing.^22^, ^23^ In our sample, anterior quadrant perforations predominated (64%), in which graft integration was attained in 96% of cases (*p*=0.430). Graft integration was 92.8% in posterior quadrants (*p*=0.348); this shows that the perforation site did not correlate significantly with surgical success.

A few studies^4^, ^24^, ^25^ have shown that the status of the opposite ear (perforation or atelectasis) was a factor that decreased surgical success rates, with lower graft integration rates when the opposite ears were diseased. Other studies,^10^, ^17^ however, found no such association. In our sample, graft integration in patients with diseased contralateral ears was 100% (*p*=0.646); graft integration was 93.9% in patients with normal contralateral ears (*p*=0.348), showing that the status of the contralateral ear was not statistically significant for surgical success.

Belluci (1973)^26^ and Kartush et al. (2002)^27^ stated that smoking was not a prognostic factor for graft integration, but that it had a significant negative longterm effect in the surgical result of patients with COM. Becvarovski et al. (2001)^28^ and Onal et al.(2005),^4^ on the other hand, have stated that smoking has a negative effect on surgical results. Becvarovski et al. reported a 60% failure rate in smoking patients, compared to 20% in non-smokers. Onal et al. also found a 52.3% failure rate in smoking patients compared to 21.3% in non-smokers.

The effects of smoking on the middle ear may be classified as local, regional, or systemic. Local effects are those on the mucociliary apparatus, such as altered viscosity or quantity of the mucus, destruction of auditory tube and middle ear hair cells, and altered ciliary function. The regional effects are those resulting from auditory tube obstruction because of nasal conditions resulting from chemical irritation. Systemic effect is the immunosuppression that may increase the susceptibility to infection.^28^

In our sample, graft integration was 100% in smokers (*p*=0.626), and 91.3% in non-smokers (*p*=0.435). Our results concur with those of Belluci and Kartush in that smoking was not statistically significant for surgical success.

Many otorhinolaryngologists believe that a dry ear is important for graft integration; others think that this is not so important for surgical success. A few studies have shown that a wet ear is a negative prognostic factor for graft integration.^18^, ^29^, ^30^, ^31^ Several other studies have found no significant statistical correlation.^4^, ^5^, ^32^ In our study, graft integration in patients with dry ears occurred in 94.5% of cases (*p*=0.254); it was 100% in patients with wet ears (*p*=0.387). Thus, the status of the ear (dry or wet) was not statistically important for surgical success.

The presence of tympanosclerotic plaques did not correlate with surgical success in Onal et al.'s (2005)^4^ study, which was a review of 74 patients that had such plaques and that underwent tympanoplasty. Pinar et al. (2008)^24^ found that absence of tympanosclerosis increases the success rate of tympanoplasty. In our study, graft integration in patients with tympanosclerotic plaques occurred in 92.3% of cases (*p*=0.412); it was 96.1% in patients without such plaques (*p*=0.456). Thus, our results concur with those of Onal et al., and show that there is no statistical significance between the presence of tympanosclerosis and surgical success.

A present, autogenous grafts such as the temporal fascia and tragus perichondrium cartilage are advocated.^33^, ^34^ Temporal fascia grafts have been seen by several authors as the gold standard, and has been the main graft material used in tympanoplasties of children and adults.^35^ Although tragus perichondrium is accepted as a graft, few studies on this material have been published. Both yield successful graft integration in about 90% of tympanoplasties.^36^ In our study, graft integration when using temporal fascia occurred in 94.2% of cases (*p*=0.231); it was 100% when tragus perichondrium was used (*p*=0.431), showing that the type of graft was not statistically important for surgical success.

Therefore, in our study, factors such as age, perforation size, the perforation site, the status of the contralateral ear, smoking, the state of the middle ear mucosa, tympanosclerosis plaques, and the type of graft did not correlate statistically with surgical success.

## CONCLUSION

The results led us to conclude that retroauricular tympanoplasties (underlay technique) should be considered in medical residency programs because:

they are easily performed;

the results - graft integration - are excellent and above the mean that is mentioned in the literature for other techniques. These results were independent of factors that several authors consider relevant;

the results in terms of improved hearing and decreased tinnitus are significant.
